# A systematic review of the association between the age of onset of spinal bulbar muscular atrophy (Kennedy's disease) and the length of CAG repeats in the androgen receptor gene

**DOI:** 10.1016/j.ensci.2024.100495

**Published:** 2024-01-28

**Authors:** Dante J. Bellai, Mark G. Rae

**Affiliations:** Department of Physiology, Western Gateway Building, University College Cork, Cork, Ireland

**Keywords:** Kennedy's disease, Spinal bulbar muscular atrophy, CAG repeat, Activities of daily living, Electromyography, Nerve conduction studies, Compound muscle action potentials, Androgen receptor gene

## Abstract

**Introduction:**

Spinal bulbar muscular atrophy (SBMA) is an X-linked recessive motor neuron disorder caused by the presence of ≥38 CAG repeats in the androgen receptor gene. Existing literature indicates a relationship between CAG repeat number and the onset age of some motor symptoms of SBMA. This review explores the effect of larger *versus* shorter CAG repeats on the age of weakness onset in male SBMA patients.

**Methods:**

Three databases (October 2021; MEDLINE, SCOPUS, and Web of Science), Cambridge University Press, and Annals of Neurology were searched. 514 articles were initially identified, of which 13 were included for qualitative synthesis.

**Results:**

Eleven of the thirteen articles identified a statistically significant inverse correlation between CAG repeat length and age of weakness onset in SBMA. Five studies indicated that SBMA patients with between 35 and 37 CAG repeats had an older age of weakness onset than patients with over 40 CAG repeats. The minimum number of CAG repeats associated with weakness was in the mid-to-late thirties.

**Conclusion:**

Identification of a relationship between CAG repeat number and age of weakness may enable earlier detection and intervention for SBMA. In the future, studies should use interviews, chart reviews, and standardized scoring methods to reduce effects of retrospective bias.

## Highlights

• SMBA is a rare, understudied illness.
• Androgen receptor gene CAG repeat number *versus* age of onset of weakness is inversely correlated.
• CAG repeat number in the mid-thirties is pathognomonic, supporting existing literature.
• This relationship may enable the early detection and/or treatment of SBMA.

## 1.1 Introduction

Spinal bulbar muscular atrophy (SBMA), also known as Kennedy's disease, is an X-linked recessive motor neuron disorder associated with >38 CAG repeats in the androgen receptor (AR) gene [1], that affects approximately 1/40,000 men (although prevalence is greater in certain locations, such as the Vaasa region of Finland) [2]. Increased CAG expansion induces toxic polyQ-AR fragments, resulting in the disruption of transcription, mitochondrial function, protein cycling, cell signaling pathways and autophagy [1,3]. The average age of onset of SBMA is in the third or fourth decade and, although female carriers display some symptoms of the condition such as distal motor deficits, cramping and/or fasciculations [1], men are more severely affected by the condition [4] with symptoms including dysarthria, dysphagia, gynecomastia, cramping, fasciculations and tremor [1].

Generally, SBMA follows a gradual progression, starting with postural tremors of the upper limb in the early thirties [5]. In addition to the onset of dysarthria and dysphagia in the forties, lower limb motor deficits also develop, eventually requiring the use of walking aids by the fifth decade [5]. Finally, patients become wheelchair-bound by, or during, their sixth decade [5]. Patients with early-stage involvement of bulbar musculature often die from recurrent aspiration pneumonia in their fifties [6].

Although it is known that the age of onset of non-motor manifestations of SBMA is independent of CAG repeat length, primary literature indicates that there is also a clear negative correlation between the age of onset of motor manifestations and CAG repeat number (for review see [4]). Although several studies [[5], [6], [7], [8], [9], [10], [11], [12], [13], [14], [15], [16], [17]] have pondered the relationship between CAG repeat number and the age of the most frequent symptom of SBMA, weakness [18], to date, no systematic reviews have specifically focused on the possible connection between these two factors. As such, herein we seek to address this deficit by exploring the effect of a greater number of CAG repeats on the age of weakness onset in comparison to a lower number of CAG repeats in male SBMA patients (between the ages of 11 and 83) who did not suffer from other major chronic illnesses.

Given that very little is known about the etiology of SBMA, probably due to its relatively low prevalence in the population, and that current treatments for SBMA only facilitate symptom management, men with the condition should be identified as soon as possible such that treatment of symptoms and counselling can begin sooner [4]. As such, any addition to the SBMA-associated literature that increases understanding of the disease is timely.

Primary literature was reviewed with the following objectives borne in mind:

1. To determine if the association between CAG repeat number and age of onset of symptoms of SBMA is significant.
2. Determine what minimum and maximum number of CAG repeats are associated with weakness onset in SBMA.

## 1.2 Methodology

### 1.2.1 Study eligibility

Inclusion criteria (see Table B.1, Appendix B) enabled the identification of as many articles as possible that addressed the relationship between CAG repeat number and the specific age of onset of weakness in SBMA patients. Selected studies must have at least some male SBMA patients, while female-only studies were excluded. Only studies that examined SBMA patients (with or without female carriers) that had no other major health conditions such as a concomitant diagnosis of schizophrenia, were included. Studies that involved patients with health conditions that commonly co-occur with SBMA (*e.g.* type II diabetes) were included. Additionally, the age profile of participants had to range from 11 to 83, which was the most common age range encountered in our literature search.

Any form of peer-reviewed, primary research study (*e.g.* case control study, *etc.*), was included if it was written in English and did not include animal subjects. However, reviews, commentaries and/or editorials were excluded. Both freely available texts and articles that could only be purchased [7] were included if they were accessible online. Given the rare nature of the disease and the relative dearth of available literature pertaining to it, no date range was applied to searches.

### 1.2.2 Study identification

**Fig. 1:**
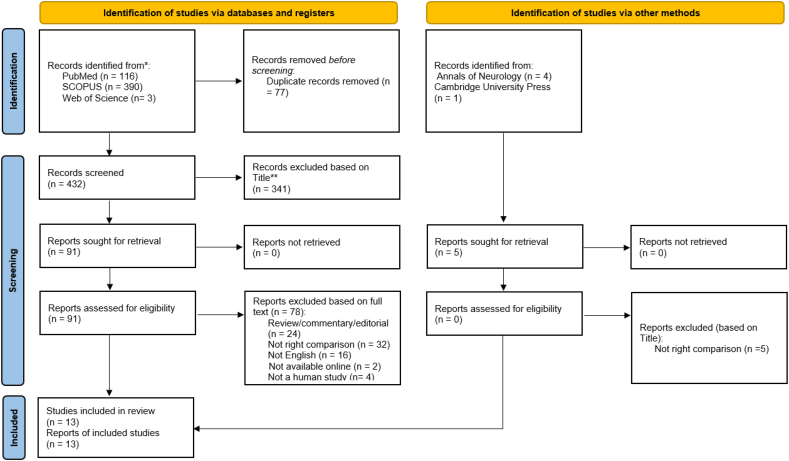
Flow chart of screening, and criteria for inclusion and exclusion of articles [8].

Searches were conducted between September and October 2021, by an independent reviewer. PubMed was subsequently searched using the following combined terms that were designed to address three concepts: “spinal bulbar muscular atrophy”, “age of onset of muscle weakness”, and “CAG repeat”, which yielded 116 results with additional searches in SCOPUS and Web of Science databases, utilizing variations of the same three concepts, which generated 390, and 3 results, respectively. Individual searches were conducted in Cambridge University Press, and the Annals of Neurology: Wiley Online Library which yielded six results (see Appendix A for list of search terms, and Fig. 1 for the search process).

### 1.2.3 Study selection

As SBMA has not been extensively investigated, we sought to find as many eligible articles as possible such that search filters were not applied. Our searches yielded a total of 515 results (Fig. 1). These were uploaded to EndNote, where duplicates were removed both using Endnote software, and manually during title sorting. Overall, 346 titles from databases were excluded, with five articles identified from individual journal searches also being excluded (Fig. 1). Titles that mentioned SBMA and clinical signs or symptoms and/or pathophysiology, as well as those that mentioned a relationship between CAG repeats and the onset of muscle weakness in male SBMA patients (with or without a female carrier population being part of the study) were included. Given the scarcity of data, all studies, irrespective of the site of onset of weakness (*e.g.* facial, bulbar or spinal) in the patients analyzed, were considered. Ultimately, 91 articles passed the initial title screening process (Fig. 1 and Table B.2 which is in Appendix B). Articles were then further screened based on their abstract and full text, with 78 articles being further excluded. In all, 13 articles met our inclusion criteria and passed screening (Fig. 1).

## 1.3 Results

### 1.3.1 Results

The study design of the 13 papers deemed eligible for inclusion in this review was as follows: ten quantitative observational [7,[9], [10], [11], [12], [13], [14], [15], [16], [17]], one quantitative longitudinal prospective [18], and two quantitative longitudinal retrospective studies [5,19]. There were four Japanese studies [5,7,9,12], one German [11], one Canadian [18], one Polish [13], one Taiwanese [19], one Italian [10], two Korean [14,17], one Chinese [16], and one American study [15]. Populations varied depending on the country in which each study took place. Sample sizes ranged between 14 and 223 patients. Table 1 and its full version, D.1, summarize the main results of each study.

### 1.3.2 Critical appraisal

As all reviewed studies were quantitative in nature, they were graded using the EBL Critical Appraisal Checklist [20] (see Appendix C), which provides an overall score for a study based upon the validity of its population, data collection, study design, and results (see Table B.3). For a study to be designated as valid, it must have an overall validity score of 75%. As shown in Table B.3, four papers failed this test [9,13,17,19] with overall scores of 67% (Doyu et al., 1992), 71% (Fu et al., 2013), 72% (Song et al., 2015) and 62% (Tomik et al., 2006), primarily due to poor population validity, but also due to poor results validity in the cases of Doyu et al. (1992) and Song et al. (2015) [9,17].

### 1.3.3 Objectives

#### 1.3.3.1 Objective 1: To determine if the association between CAG repeat number and age of onset of symptoms of SBMA is significant

All thirteen eligible studies examined the relationship between CAG repeat expansion number and the age of onset of weakness in SBMA patients [5,7,[9], [10], [11], [12], [13], [14], [15], [16], [17], [18], [19]] – and could be divided into two groups: i) studies that used a scoring system to assess weakness and other signs, and, ii) studies that used informal patient interviews and/or chart reviews only. Two studies used a combination of methods [5,14]. In the first group, scoring systems included: the activity of daily living scores [9,10,14,17], the Limb Norris score, Norris Bulbar score and ALS functional rating scale-revised [12], the Neuropathy Impairment Score [18], an unspecified standard scoring system [11], and a scale to grade weakness [7]. The second group of studies used either patient interviews [7] or both chart reviews and interviews [[13], [14], [15],^19^], or chart reviews only [16] to screen symptoms and/or signs of SBMA.

With regard to assessing CAG repeat number and the age of onset of weakness in SBMA patients (see Table 1, and Table D.1 in Appendix D), the available results indicated that there were three main outcomes: 1. No significant relationship identified either due to identifying a statistically insignificant inverse correlation [19], or a significant correlation without an r value [7], or no correlation [13]; 2. Identification of either a significant moderate inverse relationship between CAG repeats and the age of onset of weakness [5,9,10,14,[16], [17], [18]], or a strong inverse correlation [11,15] ; and 3. Identification of a relationship based upon phenotype, as with Suzuki et al. (2007), where the number of CAG repeats was compared with weakness onset in the motor-dominant phenotype [12]. Note that group 2 also contains results obtained from Lee et al. (2005), which claimed a “weak” correlation (*r* = −0.4) [14], as the correlation they identified is classified as "moderate" based on their own published r value (*e.g.,* ± 0.4 to ±0.6 is moderate, based upon accepted r value classification [21]).

**Table 1:** Summary of studies that are included in this review article.

Author (Year), Title	Objective	Key Findings / Results
Fu et al., (2013), Long-term follow-up of spinal and bulbar muscular atrophy in Taiwan	To investigate the long-term prognosis of SBMA and early diagnosis of SBMA.	1. Insignificant inverse correlation between CAG repeat number and onset of weakness. Most commonly reported symptoms included hand tremor, pectoral fasciculation and limb weakness. 2. Nerve conduction studies showed decreased amplitude of compound motor action potentials between controls and SBMA patients. 3. Functional disability was slowly progressive – only three patients became wheelchair bound. 4. Creatine kinase elevated in 17/18 patients. 5. CAG repeat range 42–53 (mean 47 ± 3).
Doyu et al., (1992), Severity of X-linked bulbospinal neuropathy correlates with size of the tandem CAG repeat in AR receptor gene	To examine the relationship between CAG repeats and age of onset of SBMA.	1. CAG repeat number significantly correlated with age of onset of limb weakness (*r* = −0.596, *p* < 0.001) and age-adjusted scored disability (*r* = 0.446, *p* < 0.03). 2. CAG repeat range, 40–55.
Sperfeld et al., (2002), X-linked bulbospinal neuronopathy	To define age of onset of SBMA in addition to determining early symptoms of SBMA	1. Correlation between CAG repeat number and age of onset of weakness (*r* = −0.65; *p* < 0.01), but not age of onset of SBMA. Weakness not an initial symptom. 2. Early symptoms included gynecomastia, premature exhaustion and muscle pain. 3. No correlation between occurrence of non-motor signs or increased creatine kinase levels, tremor or additional myopathic changes in muscle prosections. 4. CAG repeat range of 40–42.
Rosenbohm et al., (2018), The metabolic and endocrine characteristics in spinal and bulbar muscular atrophy	Systematic phenotyping of German SMBA cohort by assessing endocrine, metabolic and neuromuscular status.	1. Paresis duration and CAG repeat number correlated with dehydroepiandrosterone concentration. CAG repeat number and age of onset of weakness were inversely correlated (r = − 0.72; *p* < 0.0001). 2. Almost all patients reported muscle weakness, gynecomastia, tremor, and dysphagia. 3. Serum creatine kinase concentration did not relate to CAG repeat number or disease duration. 4. Mean CAG repeat number of 46.2 ± 3.3 (range 39–55) with mean age-at-weakness-onset of 44.4 ± 12.0 years
Mariotti et al., (2000), Phenotypic manifestations associated with CAG-repeat expansion in the androgen receptor gene in male patients and heterozygous females: a clinical and molecular study of 30 families	To report on phenotypic manifestations of SBMA in carriers and male patients.	1. Significant correlation between CAG repeat number and age at onset of weakness (*r* = −0.408; *p* < 0.02). Muscle weakness was most commonly-reported symptom. 2. Age at onset of weakness differed by >20 years in patients expressing identical CAG expansion numbers. 3. Increased CAG expansion associated with a younger age at onset. No significant correlations between CAG repeat number or disability scores. 4. Mild bulbar motor deficits (*e.g.* tongue fasciculations found in carrier women). 5. CAG repeat range of 39–50.
Sinnreich et al., (2004), Neurologic course, endocrine dysfunction and triplet repeat size in spinal bulbar muscular atrophy	Investigate the role of diabetes, CAG expansion length and gynecomastia as factors that modify the neurologic aspects of the expression of SBMA.	1. CAG repeat number and endocrine diseases did not correlate with the rate of progression of SBMA or neurologic involvement of the disease. 2. Statistically significant correlation between CAG repeat number and age of onset of weakness (*r* = −0.53, r^2^ = 29%, *p* = 0.01). 3. CAG repeat range from 36 to 55.
Suzuki et al., (2007), CAG repeat size correlates to electrophysiological motor and sensory phenotypes in SBMA	To clarify sensory and motor nerve components of SBMA using nerve conduction studies in addition to correlating CAG repeat number with electrophysiological phenotypic dominancy.	1. CAG repeat number and age at onset differed depending on dominant phenotype (*e.g.* longer CAG repeats more closely linked with motor phenotype). Greater age of onset and age at examination observed in patients with shorter CAG repeat number (*p* < 0.0001). 2. CMAP amplitude decreased in SBMA patients and increased in patients with larger CAG repeat number (>/47). 3. Mean CAG repeat number in AR gene was 47.8 ± 3.1, with range of 41–57.
Shimada et al., (1995), X-linked recessive bulbospinal neuronopathy: clinical phenotypes and CAG repeat Size in androgen receptor gene	To assess clinical phenotypes and CAG repeat number of androgen receptor gene in 96 SBMA patients.	1. Decreased serum creatine kinase concentration, muscle strength, ADL scores dependent on both age and duration. 2. Correlations present between age of weakness onset and CAG repeat number (*p* < 0.0001). 3. Correlation between CAG repeat number and gynecomastia (*p* < 0.05). No correlation between either presence or absence of this condition and age of onset/duration. Same for diabetes mellitus. 4. CAG repeat range, 41–52.
Tomik et al., (2006), A phenotypic-genetic study of a group of Polish patients with spinal and bulbar muscular atrophy	Phenotype-genotype correlation in Polish SBMA males and heterozygous female carriers.	1. No correlation between CAG repeat number and either duration of disease or age of onset of weakness. Phenotypic expression in SBMA may not depend entirely on CAG repeat number. 2. Male patients had common signs of progressive hand tremor, slight dysphagia, nasal voice, gynecomastia, decreased potency, distal limb weakness, facial muscular weakness and orofacial fasciculations. 3. One carrier presented with minimal distal weakness, leg cramping and history of fasciculations. 4. Mean range of CAG repeat number of 45–52 in males, and 46–68 in females. 5. CAG repeat range, 45–52.
Lee et al., (2005), Phenotypic variability in Kennedy's disease: implication of the early diagnostic features	To determine frequency of most common clinical features of Kennedy's disease in addition to early symptoms of SBMA.	1. Perioral fasciculation with bulbar paresis, elevated creatine kinase levels, hand tremor, hyporeflexia, and limb weakness with wasting were most consistent clinical findings. 2. Half of cases suffered from gynecomastia, sensory abnormalities, and family history of SBMA. 3. Weak correlation between CAG repeat number and age of onset of paresis (*r* = −0.4). Age of onset of other symptoms and CAG repeat number were not significantly correlated. 4. CAG repeat number, 45–52.
Atsuta et al., (2006), Natural history of spinal and bulbar muscular atrophy (SBMA): a study of 223 Japanese patients.	Investigate natural course of SBMA as assessed by 9 ADL milestones in 223 Japanese SBMA patients, and correlate this with age of onset of specific milestones during course of disease with the CAG-repeat number in AR gene.	1. Ages at onset of each ADL milestone strongly correlated with CAG repeat number in AR gene. Inverse correlation between onset of muscle weakness and CAG repeat number (R = -0.483, *p* < 0.001). 2. CAG repeat number did not correlate with time intervals between each ADL milestone. 3. Levels of serum testosterone maintained at relatively high levels even at advanced ages. 4. Mean CAG repeat number of 46.6 ± 3.5, with range of 40–57.
Ni et al., (2015), Genotype-phenotype correlation in Chinese patients with spinal and bulbar muscular atrophy	Elucidate genotype-phenotype correlation among Chinese SBMA patients.	1. Inverse correlation between CAG repeat number and age of onset of muscle weakness R^2^ = 0.34, *p* < 0.0001. 2. Average CAG repeat number of 48.6 ± 3.5. 3. Inverse correlation between creatine kinase level and disease duration, and age at examination (*p* = 0.019 and *p* = 0.004, respectively). Additionally, all nerve conduction findings, except the amplitudes of median nerve compound motor action potential, were positively correlated with CAG repeat number. 4. Average CAG repeat number of 48.6 ± 3.5 (range 42–61), with average age of onset of muscle weakness of 44.2 ± 10.2 (24–71) years.
Song et al.*,* (2015), Clinical characteristics and genotype-phenotype correlation of Korean patients with spinal and bulbar muscular atrophy	Genotype-phenotype comparison of clinical and genetic data from Korean SBMA and control patients from different ethnicities.	1. Significant inverse correlation between CAG repeat number and age of onset of symptoms, including muscle weakness (r = −0.407, *p* = 0.009). 2. Median ages of onset and age at diagnosis were 44.5 and 52.5 years, respectively, and median CAG repeat number of 44 (range 39–55). 3. Median interval between median rate of disease progression and onset and diagnosis were 0.23 score/year and 5.0 years respectively.

#### 1.3.3.2 Objective 2: To determine what minimum and maximum number of CAG repeats are associated with weakness onset in SBMA

As shown in Table 1 (and Table D.1 in Appendix D), although all eligible studies did report the number of CAG repeats, they differed based upon the way in which said data was presented such that they can be organized into three main groups: 1. Five studies that listed the ages of participants alongside the minimum and maximum number of recorded CAG repeat number in a retrievable form [7,9,10,14,18]; 2. Seven studies that listed CAG repeat number and age ranges [5,11,13,[15], [16], [17],^19^]; and, 3. One study that involved a unique experimental design [12].

In the first group, the article by Sinnreich et al. (2004) reported the shortest number of CAG repeats at 36 (weakness onset at age 46), ranging up to 55 (weakness onset at 38), with the cut-off for consideration being >35 repeats [18]. Mariotti et al. (2000) also reported a minimum CAG repeat number in the thirties, ranging from 39 to 50, with the patient with the fewest repeats experiencing weakness at age 50, and the two patients with the most repeats experiencing weakness at ages 35 and 31 respectively – while indicating that a normal CAG repeat range was between 12 and 30, and that in SBMA it could be from 39 to 62 [10]. Of these five studies, three reported a minimum number of CAG repeats ≥40: Doyu et al. (1992), who reported a range of 40–55, noting that the patient with the highest number of repeats experienced weakness onset at age 25, forty-five years before the patient with the lowest number of repeats (at age 70) [9]; Shimada et al. (1995), who identified a slightly later age-at-weakness-onset (age 49) with a shorter repeat of 41 compared with a patient with the greatest CAG repeat number, 52, and the onset of weakness at age 44 [7]; and, lastly, Lee et al. (2005) who reported the highest number of minimum CAG repeats, 45, and a maximum repeat number of 54, with an age-at-weakness-onset of 55 and 39 respectfully [12].

In the second group of studies, the articles by Song et al. (2015) and Rosenbohm et al. (2018) both reported the lowest minimum CAG repeat number of the group at 39 [11,17]. However, their reported age ranges and average CAG repeat number differed, with Song et al. (2015) reporting a mean of 44 CAG repeats (39–55) and an average age-at-onset of symptoms of SBMA of 44.5 (20.0–71.0) years [17], while Rosenbohm et al. (2018) reported a mean length of 46.2 ± 3.3 (39.0–55.0), and a mean age-at-weakness-onset of 44.4 ± 12.0 (25.0–75.0) years [11]. Three of the seven studies reported a minimum number of CAG repeats of exactly 40. Specifically, Sperfeld et al. (2002) proposed that the threshold of normal CAG repeat number is 38 and reported a range of 40–42 CAG repeats [15]. Tomik et al. (2006) identified a CAG repeat number range of 45–52, claiming that the normal number of CAG repeats was between 5 and 33, but that for SBMA, it was between 40 and 62 [13]. Atsuta et al. (2006) only reported a CAG repeat number range of 40–57 for SBMA, with a mean number of 46.6 ± 3.5 [5]. Finally for this group, the two studies with the highest recorded minimum CAG repeat number were Ni et al. (2015), with an average number of CAG repeats of 48.6 ± 3.5 (42–61), and an average age of weakness onset of 44.2 ± 10.2 (24–71) years [16], and Fu et al. (2013), who found a CAG repeat range of 42–53 (mean 47 ± 3) [19]. The latter group proposed a normal CAG repeat number range between 14 and 32, with >40 repeats being reported as abnormal [19].

One study, by Suzuki et al. (2007), differed from both aforementioned groups. This study subdivided patients with a CAG repeat number < 47 (short repeats) from those with a repeat number ≥ 47 and then conducted electromyographic and nerve conduction studies on both groups separately [12]. They reported a mean CAG repeat number of 47.8 ± 3.1, with a range of 41–57 CAG repeats [12].

## 1.4 Discussion

### 1.4.1 Discussion of results

CAG repeat number may account for approximately 60% of clinical heterogeneity in SBMA patients, with genetic, environmental and epigenetic factors also likely to play some role in influencing disease progression [1]. However, the contribution of these relative unknowns aside, determining the correlation between the most commonly-mentioned SBMA symptom, weakness, and the primary clinical marker for the condition, CAG repeat number on the AR gene, might potentially uncover a strong indicator of disease onset that could enable early SBMA detection and, thereafter, management. As such, this review is important and timely given that no other review to date has specifically investigated a possible connection between the age of onset of weakness in SBMA and CAG repeat number.

All of the studies utilized for this review addressed our first objective, with 11 of the 13 indicating that a significant inverse correlation did indeed exist between CAG repeat number and onset of SBMA weakness [5,7,[9], [10], [11],[15], [16], [17], [18]] (Table 1). With regard to this objective, our review presented two major issues. Firstly, a surprising number of articles failed screening. Although the study by Doyu et al. (1992) only just failed our initial screening due to poor population validity (56%), data collection validity (63%) and results validity (67%) (overall score 67%) (Table B.3, Appendix B) [9], we felt that the use of disability scores to assess clinical severity in this paper, combined with the limited number of studies available, mitigated against its exclusion. Notably, a single study did find that shorter CAG repeat number (<47) was associated with an *older* onset of weakness [12].

**Table B.3:** Validity score for quantitative studies using the EBL critical appraisal checklist.

Study	Population Validity %	Data collection Validity %	Study Design Validity %	Results Validity %	Overall Score %
Fu et al.*,* (2013)	56	63	80	83.3	71
Doyu et al., (1992)	56	63	80	67	67
Sperfeld et al., (2002)	83.3	75	80	83.3	80.4
Rosenbohm et al., (2018)	78	75	100	83.3	84
Mariotti et al., (2000)	83.3	75	80	83.3	80
Sinnreich et al., (2004)	83.3	75	100	83.3	85.4
Suzuki et al., (2007)	67	100	100	83.3	88
Shimada et al., (1995)	67	63	80	100	78
Tomik et al., (2006)	33	50	80	83.3	62
Lee et al., (2005)	67	86	80	83.3	79.1
Atsuta et al., (2006)	67	57	100	83.3	77
Ni et al., (2015)	67	60	100	83.3	78
Song et al., (2015)	50	86	100	50	72

Interestingly, of the three studies that failed to find a strong, or even a moderate, statistically significant correlation between CAG repeat number and age of onset of weakness, two had poor external validity [13,19]. For example, the overall validity for the article by Tomik et al. (2006) was only 62%, primarily due to its poor population validity (33%; Table B.3, Appendix B), resulting mainly from the lack of age-matched healthy controls to compare EMG results against, as well as the small sample size (11 SBMA patients and three carriers, see Table D.1 in Appendix D). Similarly, the study by Fu et al. (2013), also had a relatively low overall validity score of 71%, also mostly due to its poor external validity (50%) (Table B.3) due to its small population size and retrospective nature [19]. Although the third study that did not find a statistically significant correlation, by Lee et al. (2005), did pass screening validity with an overall validity score of 78% it also had a low population validity score (60%; Table 1 and Table D.1) stemming from its small sample size [14]. Other articles that failed screening included Song et al. (2015), who reported only a moderate correlation between CAG repeat length and SBMA weakness (overall score 72%; Table 1 and Table D.1), derivative of both poor population and results validity (both 50%) due to sample size issues and a lack of accountability for confounding factors [17].

Our review of included articles also revealed a second major issue: the need for larger scale studies to better characterize SBMA. Indeed, Fu et al. (2013) suggested that any future characterization of the functional progression, and the clinical and electrophysiological aspects of SBMA, be conducted using larger scale prospective studies [19]. Similar sentiments were expressed by Song et al. (2015) and Lee et al. (2005) [14,17].

With regard to our second objective, the majority of studies presented both CAG repeat number data and ages of onset of weakness, such that these data could be compared across studies to determine the minimum and maximum number of repeats at age of weakness onset in SBMA [7,9,10,14,18], apart from two articles [11,17]. Of the five studies that addressed the objective, CAG repeat number ranged from the mid-thirties to the mid-fifties, coinciding with the accepted marker of ≥38 repeats used to indicate the presence of SBMA [1], with all five studies claiming that patients with larger repeat numbers had an earlier onset of weakness [7,9,10,14,18]. There has been some data collected with regard to the effect of the number of CAG repeats, with median lengths varying in various racial groups (as reported by the Neuromuscular Disease Center at Washington University): White Caucasian (21−22), African American (19–20), Hispanic (23) and Asian (22−23) [22]. They state the CAG repeat range for SBMA to be 40–68 (closely approximating the ranges reported in this review) while claiming that the CAG repeat number is associated with earlier and more severe disease onset but not specific clinical features [22].

### 1.4.2 Future considerations

We believe that any future SBMA studies investigating a possible connection between CAG repeat number and age of weakness onset should consider the potential effects of mosaicism as this may help to explain atypical presentations of SBMA (*i.e.* a patient with 26 CAG repeats and symptoms of SBMA) [13]. Larger sample sizes would also be desirable in order to enable more accurate measurement of the decline in motor function in SBMA patients over time, a claim repeated by multiple articles (Table D.1, Appendix D). Furthermore, age-matched controls and/or controls with other muscular atrophies should be included to provide appropriate baseline comparison data during electromyographic or nerve conduction studies. Finally, one could also mitigate against error due to the use of a variety of methods to assess function in SBMA patients (complicating inter-study comparisons) by minimizing recollection bias when reporting symptoms by combining patient interview data, chart reviews and standardized scoring systems.

### 1.4.3 Limitations

One limitation of this review was its language restriction (English) such that sixteen non-English SBMA articles did not pass screening (Fig. 1). Another limitation was the inclusion of articles that were only available online, which meant that two potentially useful studies were excluded from our analysis (Fig. 1). In addition, many of the articles that were included were of Asian origin, which could arguably have introduced an ethnic genotype bias, potentially impacting the external validity of the study. However, one must note that SBMA is a rare illness, such that few institutions are likely to have the resources available to justify its study.

Finally, four articles that failed initial screening [9,13,17,19] were included in this review. Studies that used only chart reviews and/or patient interviews, but not an objective scoring system to assess symptoms [7,[13], [14], [15], [16],^19^], are subject to recollection bias, which may have impacted the recorded age at weakness onset and the correlation of interest.

## 1.5 Conclusion

Weakness is the most commonly complained of symptom among SBMA patients [1]. This review of the relevant SBMA literature has shown that the majority of studies investigating this facet of SBMA pathophysiology have demonstrated an inverse correlation between the number of CAG repeats and the age of weakness onset in SBMA patients. Furthermore, most of the aforementioned studies have proposed that the minimum number of CAG repeats associated with SBMA-linked weakness falls within at least the mid-to-late thirties range or greater. By exploring and revealing the relationship between this reliable marker of SBMA and a common clinical sign of the condition as we have done here, this review not only adds to the relatively scant literature on SBMA, but also, significantly, may facilitate earlier detection of the condition and thereby facilitate its prompt treatment.

## CRediT authorship contribution statement

**Dante J. Bellai:** Writing – review & editing, Writing – original draft, Validation, Resources, Project administration, Methodology, Investigation, Formal analysis, Data curation, Conceptualization. **Mark G. Rae:** Writing – review & editing, Visualization, Validation, Supervision.

## Declaration of competing interest

None.

This research did not receive any specific grant from funding agencies in the public, commercial, or not-for-profit sectors.

## Figures and Tables

**Table B.1:** Inclusion/exclusion criteria.

	Inclusion	Exclusion
Intervention	Studies that specifically compared CAG repeat expansion with the onset of muscle weakness in SBMA patients	Studies that addressed activities of daily living but did not specifically compare the onset of weakness with CAG repeat expansion
Population	Male SBMA patients only, or mixed male SBMA patients and female carrier patient populations aged 11–83 with SBMA	Studies that were specific to female carriers
Participants	Male SBMA patients (with or without female carriers) that had no other major health conditions or that had health conditions that commonly co-occur with SBMA such as type II diabetes	SBMA patients (with or without female carriers) that had other major health problems such as schizophrenia
Outcomes	Studies that quantified the number of CAG repeats and compared this with the age at which weakness of any kind (upper limb, lower limb *etc.*) was first reported	Studies that identified the relationship between CAG repeats and age at onset as being defined as the age at which any symptoms appear (not specifically weakness)
Date	Given that it is a rare disease, any study may be included no matter how old	N/A
Language	Studies written in English	Studies that were not written in English
Types of publication	Any kind of primary literature including: RCTs, longitudinal, case-control, mixed methods studies, observational quantitative, *etc.*	Review articles / commentaries / editorial articles
Availability	Both freely available full texts and pay-for-view articles	Articles that were not available online
Peer reviewed	Studies published in peer-reviewed journals	Studies not published in peer-reviewed journals

**Table B.2:** Excluded studies from databases based on abstract/full text.

Full text not in English	16
Did not explicitly compare age of onset of weakness with CAG repeat length in SBMA patients	32
Review/commentary/editorial paper	24
Not available online	2
Not a human study	4
Total excluded	78

**Table:** 

EBL Critical Appraisal Checklist		Atsuta et al. (2006)	Ni et al. (2015)	Song et al. (2015)	
Section A: Population	Is the study population representative of all users, actual and eligible, who might be included in the study?	Y	U	N	
	Are inclusion and exclusion criteria definitively outlined?	Y	Y	Y	
	Is the sample size large enough for sufficiently precise estimates?	Y	Y	Y	
	Is the response rate large enough for sufficiently precise estimates?	Y	Y	Y	
	Is the choice of population bias-free?	U	U	N	
	If a comparative study: Were participants randomized into groups? Were the groups comparable at baseline? If groups were not comparable at baseline, was incomparability addressed by the authors in the analysis?		N/A	N/A	
		N			
		U			
		Y			
	Was informed consent obtained?	Y	Y	U	
Section B: Data Collection	Are data collection methods clearly described?	Y	Y	Y	
	If a face-to-face survey, were inter-observer and intra-observer bias reduced?	N/A	N/A	N/A	
	Is the data collection instrument validated?	Y	U	Y	
	If based on regularly collected statistics, are the statistics free from subjectivity?	N	N/A	Y	
	Does the study measure the outcome at a time appropriate for capturing the intervention’s effect?	Y	Y	Y	
	Is the instrument included in the publication?	Y	N/A	Y	
	Are questions posed clearly enough to be able to elicit precise answers?	U	Y	Y	
	Were those involved in data collection not involved in delivering a service to the target population?	N	U	N	
Section C: Study Design	Is the study type / methodology utilized appropriate?	Y	Y	Y	
	Is there face validity?	Y	Y	Y	
	Is the research methodology clearly stated at a level of detail that would allow its replication?	Y	Y	Y	
	Was ethics approval obtained?	Y	Y	Y	
	Are the outcomes clearly stated and discussed in relation to the data collection?	Y	Y	Y	
Section D: Results	Are all the results clearly outlined?	Y	Y	Y	
	Are confounding variables accounted for?	Y	Y	N	
	Do the conclusions accurately reflect the analysis?	Y	Y	Y	
	Is subset analysis a minor, rather than a major, focus of the article?	U	N	U	
	Are suggestions provided for further areas to research?	Y	Y	Y	
	Is there external validity?	Y	Y	N	
**Calculation for section validity: (Y+N+U=T)** If Y/T <75% or if N+U/T > 25% then you can safely conclude that the section identifies significant omissions and that the study’s validity is questionable. It is important to look at the overall validity as well as section validity.	**Calculation for overall validity: (Y+N+U=T)** If Y/T ≥75% or if N+U/T ≤ 25% then you can safely conclude that the study is valid.				
**Section A validity calculation: 1/ 4= 25% not valid** **Section B validity calculation: 5/ 7= 71% not valid** **Section C validity calculation: 1/ 5= 20% not valid** **Section D validity calculation: 4/ 6= 67% not valid**	**Overall validity calculation: 11/ 22 = 50% not valid**				

**Table D.1:** Characteristics of included studies.

Author (Year), Title	Objective	Study Type, Population, Sample Size, Country	Study Methodology	Key Findings	Strengths and Limitations	Future Directions
Fu et al., (2013), Long-term follow-up of spinal and bulbar muscular atrophy in Taiwan	To investigate the long-term prognosis of SBMA and early diagnosis of SBMA.	1. Quantitative longitudinal retrospective. 2. Male Taiwanese SBMA patients from Chang Gung Memorial Hospital in Linkou, Taiwan with mean age of 43 years. 3. 21 patients from 14 families. 4.Taiwan	Patients selected from 1993 to 2010 and studied through patient interviews and chart reviews to determine onset of age of symptoms and signs of SBMA like weakness. Genomic DNA extracted to measure number of CAG repeats. The number of CAG repeats on exon 1 of the androgen receptor was determined using polymerase chain reaction. The correlation between the age of onset of weakness and the lengths of CAG repeats were analyzed using linear regression and Pearson's correlation coefficient (where *p* < 0.05 was significant). Serum creatine kinase in addition to other proteins also measured. Nerve conduction studies of median, ulnar and sural nerves performed on SBMA patients and 20 healthy controls.	1. Insignificant inverse correlation between number of CAG repeats and onset of weakness. Most commonly reported symptoms included hand tremor, pectoral fasciculation and limb weakness. 2. Nerve conduction studies showed decreased amplitude of compound motor action potentials between controls and SBMA patients. 3. Functional disability was slowly progressive – only three patients became wheelchair bound. 4. Creatine kinase elevated in 17/18 patients. 5.CAG repeat range 42–53 (mean 47 ± 3).	Authors indicated study was limited by its retrospective nature and limited sample size.	Authors recommended that, in future, larger sample sizes could be used in prospective studies to clarify the functional progression, in addition to the clinical and electrophysiological decline of SBMA patients.
Doyu et al., (1992), Severity of X-linked bulbospinal neuropathy correlates with size of the tandem CAG repeat in AR receptor gene	To examine the relationship between CAG repeats and age of onset of SBMA.	1. Quantitative observational. 2. Members of 21 Japanese families with confirmed SBMA. Age range of 31–83 at examination. 3. 26 patients 4.Japan	Patients were selected from 21 available pedigrees and were screened for first symptoms including limb weakness. Scored using an activity of daily living scoring system. Genomic DNA was extracted and PCR performed to measure the number of CAG repeats, which were then correlated with weakness and other symptoms. Specifically, PCR was conducted using an oligonucleotide located 46 base pairs upstream to the (CAG)n site, while the statistical analysis was conducted using Spearman's correlation.	1. Number of CAG repeats significantly correlated with age of onset of limb weakness (r = −0.596, *p* < 0.001) and age-adjusted scored disability (*r* = 0.446, *p* < 0.03). 2.CAG repeat range, 40–55.	Authors did not provide any strengths or weaknesses.	No further comment from the authors.
Sperfeld et al., (2002), X-linked bulbospinal neuronopathy	To define age of onset of SBMA in addition to determining early symptoms of SBMA	1.Quantitative observational. 2.Male patients from a neuromuscular outpatient clinic. Average age at examination was 48.9 years. 3. 34 patients with genetically proven SBMA. 4. America	Medical history taken and medical records reviewed. 30 patients had genomic DNA extracted and amplified using PCR to identify number of CAG repeats, then correlated with symptoms of onset (using Pearson product moment correlation, where *p* < 0.05 is statistically significant). To calculate the number of repeats that were cloned and sequenced, the polymerase chain reaction was used as product of a reference and this reference product was used in every single examination. Creatine kinase and blood glucose also measured. Nerve conduction studies performed on sural and radial nerves. Electromyography performed in one proximal and one distal muscle in at least 1 extremity. Measurement of magnetic evoked action potentials. Muscle biopsies taken from 11 patients.	1.Correlation between CAG repeat number and age of onset of weakness (r = −0.65; *p* < 0.01), but not age of onset of SBMA. Weakness not an initial symptom. 2. Early symptoms included gynecomastia, premature exhaustion and muscle pain. 3. No correlation found between occurrence of non-motor signs in addition to creatine kinase levels, tremor and additional myopathic changes in muscle prosections. 4.Adolescent onset is possible. 5.CAG repeat range of 40–42.	Authors did not provide any strengths or weaknesses.	No further comment from the authors.
Rosenbohm et al., (2018), The metabolic and endocrine characteristics in spinal and bulbar muscular atrophy	Systematic phenotyping German SMBA cohort by assessing endocrine, metabolic and neuromuscular status.	1. Quantitative observational.. 2. Male German patients with confirmed SBMA from neuromuscular referral centers in Universities of Berlin, Bochum, Dresden, Halle, Hannover, Rostock, Ulm, and Würzburg. Age range was 20–80. 3. 80 SBMA patients and 29 healthy male controls used in MRI study. 4.Germany	Standardized questionnaire addressing presentation of symptoms and family history. Blood tests performed analyzing blood glucose, lipid, and hormone levels. MRI studies conducted to examine adipose density of 11 SBMA patients and 29 healthy controls. Over 38 repeats was diagnostic for SBMA using PCR based fragment analysis. The relationship between weakness and CAG repeats for instance was analyzed using linear regression and Pearson's correlation coefficient, where statistical significance was *p* < 0.05.	1. Paresis duration and CAG repeat number correlated with dehydroepiandrosterone concentration. CAG repeat number and age of onset of weakness were inversely correlated (*r* = − 0.72; *p* < 0.0001). 2. Almost all patients reported muscle weakness, gynecomastia, tremor, and dysphagia. 3. Serum creatine kinase concentration did not relate to CAG repeat number or disease duration. 4. Mean CAG repeat number was 46.2 ± 3.3 (range 39.0–55.0) and mean age-at-weakness-onset was 44.4 ± 12.0 years	Authors indicated limitations such as recollection bias during patient interviews and absence of an age-controlled group (this could be with other muscular atrophy patients or simply healthy subjects) given that lab values are age- and muscle mass- dependent.	Controls could be used in laboratory studies so as to account for the age- and muscle mass- dependent effects on laboratory values. In addition, further studies could be conducted to evaluate the relationship between CAG repeat and weakness duration. For instance, Insulin-like growth factor binding protein 3 (IGF1BP3) should be evaluated with a larger cohort as only weak correlation with both CAG repeat length and weakness duration.
Mariotti et al., (2000), Phenotypic manifestations associated with CAG-repeat expansion in the androgen receptor gene in male patients and heterozygous females: a clinical and molecular study of 30 families	To report on phenotypic manifestations of SBMA in carriers and male patients.	1.Quantitative observational. 2. Confirmed SBMA patients and heterozygous female carriers in Italy. Male patients between 31 and 77 years. Pool of patients from 30 unrelated families. 3. 19 female carriers and 40 genetically- confirmed male SBMA patients (32/70 did not have CAG expansion). 4.Italy	DNA extracted from peripheral blood and CAG expansion determined by PCR. Serum levels of creatine and various hormones measured. Relationship between CAG and age of onset determined by linear regression analysis (Spearman's correlation test). A protocol to indicate severity of clinical disability was used and an activity of daily living score calculated for each symptom. Standard neurophysiological studies performed on carrier women.	1. Significant correlation between number of CAG repeats and age at onset of weakness (r = −0.408; *p* < 0.02). Muscle weakness was most commonly-reported symptom. 2. Age at onset of weakness differed by >20 years in patients expressing identical CAG expansion numbers. 3. Increased CAG expansion associated with a younger age at onset. No significant correlations between CAG repeat number and disability scores. 4. Mild bulbar motor deficits (*e.g.* tongue fasciculations found in carrier women). 5. CAG repeat range of 39–50.	Authors did not provide any strengths or weaknesses.	Authors did not provide any future considerations.
Sinnreich et al., (2004), Neurologic course, endocrine dysfunction and triplet repeat size in spinal bulbar muscular atrophy	Investigate the role of diabetes, CAG expansion length and gynecomastia as factors that modify the neurologic aspects of the expression of SBMA.	1. Quantitative longitudinal prospective. 2. Male patients with genetically confirmed SBMA (CAG repeats over 35) at Mayo Clinic, Rochester, MN between the ages 19–75. 3. 20 male patients 4. Canada	Neurologists conducted clinical examinations. Neuropathy Impairment Score (NIS) used to score muscle movements such as hip flexion, and score symptoms such as muscle weakness. Diabetes determined by fasting glucose measurements of above 126 mg/dL. Gynecomastia assessed in 17 patients *via* clinical examination. Bivariate correlation coefficients used to examine disease progression, CAG repeat number (where repeats >35 were included), *etc*.…	1. CAG repeat number and endocrine diseases did not correlate with the rate of progression of SBMA or neurologic involvement of the disease. 2. Statistically significant correlation between number of CAG repeats and age of onset of weakness (*r* = −0.53, r2 = 29%, *p* = 0.01). 3.CAG repeat range from 36 to 55.	Authors did not provide any strengths or weaknesses.	Authors indicated that a larger sample size could be used such that a future study would not be underpowered and perhaps able to detect whether a relationship between CAG repeats and gynecomastia and rate of disease progression could be due to confounding factors. Also indicated that more work was required to identify specific disease modifiers.
Suzuki et al., (2007), CAG repeat size correlates to electrophysiological motor and sensory phenotypes in SBMA	To clarify sensory and motor nerve components of SBMA using conducting nerve conduction studies in addition to correlating CAG repeat number with electrophysiological sensory or motor dominancy.	1.Quantitative observational. 2. Japanese males with confirmed diagnosis of SBMA. Patients between 31 and 75 years. 3. 106 patients with confirmed diagnosis of SBMA and 85 normal male control subjects. 4.Japan	Limb Norris score, Norris Bulbar score and ALS functional rating scale-revised (ALSFRS-R) scales used to assess patient motor function. Motor and sensory nerve conduction studies of median, ulnar, sural, and tibial nerves. Compound muscle action potentials (CMAPs) recorded and compared to age-matched volunteer controls. Blood extracted and CAG repeat number measured with PCR. The size of the CAG repeat was analyzed on Fraglys software version 2.2 by comparison to co-electrophoresed PCR standards with known repeat sizes described previously in Doyu et al. (1992). Pearson's correlation coefficients were used where *r* > 0.4 and *p* < 0.05 indicated statistical significance. Immunohistochemical analysis of cytoplasmic inclusions in control and SBMA primary sensory and primary motor neurons performed.	1. CAG repeat size and age at onset differed depending on dominant phenotype: Longer CAG repeats more closely linked with motor phenotype, whereas shorter CAG repeats linked with sensory phenotype. Greater age of onset and age at examination observed in patients with shorter CAG repeat number (*p* < 0.0001). 2. CMAPs decreased in SBMA patients relative to controls. 3. CMAPs increased in patients with greater CAG repeat number (>/47). 4.Mean CAG repeat number in AR gene was 47.8 ± 3.1, with range of 41–57.	Authors did not provide any strengths or weaknesses.	Authors did not provide any future recommendations.
Shimada et al., (1995), X-linked recessive bulbospinal neuronopathy: clinical phenotypes and CAG repeat Size in androgen receptor gene	To assess clinical phenotypes and CAG repeat number of androgen receptor gene in 96 SBMA patients.	1.Quantitative observational. 2. Japanese male and female patients between 11 and 83 years. 3. 95 patients from 63 families. 4.Japan	Clinical symptoms (*e.g.* limb muscle weakness) were screened for analysis, in addition to levels of creatine kinase and other substances (screened by a blood test). Onset of weakness (*e.g.* difficulty using stairs) taken as onset of disease. Severity of muscle weakness scored on deltoid, biceps brachii, iliopsoas and quadriceps femoris muscles using a grading system (0–5). Mobility of daily living designated as activity of daily living score (0–3). Awareness of dysphagia and gynecomastia also reported. Glucose tolerance tests used to assess diabetes. Genomic DNA extracted from SBMA and controls, with PCR (completed using a 21-base-pair (bp) oligonucleotide located 46 bp upstream to the (CAG)n site) used to determine CAG repeat number. The relationship between scored / measured clinical phenotypes and the CAG repeats were statistically analyzed using nonparametric Mann-Whitney *U* test or Spearman's correlation.	1. Decreased serum creatine kinase concentration, muscle strength, ADL scores dependent on both age and duration. 2. Correlations present between age of weakness onset and CAG repeat number (*p* < 0.0001). 3. Correlation between CAG repeat number and gynecomastia (*p* < 0.05). No correlation between either presence or absence of this condition and age of onset/duration. Same for diabetes mellitus. 4. Correlation between CAG repeat number if muscular weakness and ADL scores adjusted by age. 5. CAG repeat range, 41–52.	Authors did not provide any strengths or weaknesses.	Authors indicated desire to elucidate intergenerational transmission mechanism of biased paternal transmission of CAG repeat expansion in SBMA as relationship in this study was unclear.
Tomik et al., (2006), A phenotypic-genetic study of a group of Polish patients with spinal and bulbar muscular atrophy	Phenotype-genotype correlation in Polish SBMA males and heterozygous female carriers.	1.Quantitative observational. 2. Polish men with SBMA phenotypes between 20 and 70 years and female Polish carriers of SBMA between 19 and 74 years. All participants from same region. 3. 11 men with SBMA and 3 female carriers included in study. 4.Poland	11 men with clinically diagnosed SBMA and 3 female carriers recruited from cohort of 632 adult-onset motor neuron disease patients. Medical documentation reviewed, and detailed medical history taken for each patient. Electromyography and nerve conduction studies performed on eight male patients. EMG performed on one distal and one proximal muscle that was part of one lower and upper extremity at least. Reference values from literature were used. Genomic DNA extracted and PCR used to amplify CAG repeat segments of AR gene. Serum creatine kinase, glucose, and various hormones measured. Correlations between CAG repeat number and age of symptom onset (*i.e.* weakness) determined using the Pearson product moment correlation where *p* < 0.05.	1.No correlation between CAG repeat number and either duration of disease or age of onset of weakness. Phenotypic expression in SBMA may not depend entirely on CAG repeat number. 2. Male patients had common signs of progressive hand tremor, slight dysphagia, nasal voice, gynecomastia, decreased potency, distal limb weakness, facial muscular weakness and orofacial fasciculations. 3. One carrier presented with minimal distal weakness, leg cramping and a history of fasciculations. 4. Mean range of CAG repeat number of 45–52 in males, and 46–68 in females. 5. CAG repeat range, 45–52.	Authors did not provide any weaknesses. Authors suggest using participants from same region could be advantageous due to experiencing similar genetic and environmental factors.	One male patient with symmetrical, distal initial weakness, bulbar presentation, gynecomastia, and decreased potency did not have an expanded CAG sequence indicating possible mosaicism. Authors recommended that he be tested again for CAG repeat number.
Lee et al., (2005), Phenotypic variability in Kennedy's disease: implication of the early diagnostic features	To determine frequency of most common clinical features of Kennedy's disease in addition to early symptoms of SBMA.	1.Quantitative observational. 2. Male Korean SBMA patients from 17 families between 32 and 70 years. 3. 18 Male patients. 4.Korea	Patients recruited from neuromuscular clinics in Korea. CAG repeat expansion confirmed using PCR expansion of genomic DNA (where primers designed to amplify the CAG repeat region in the first exon of AR gene and each product was sequenced to determine the exact number of CAG repeats). Age at onset of each symptom evaluated by taking patient history and examination of medical records. Recording of early symptoms and signs of SBMA. Limb power assessed by Medical Research Council Scale. Distal muscles also examined for weakness using an activity of daily living score (0–3). Bulbar symptoms similarly graded. Recording of gynecomastia, serum creatine kinase, fasting glucose and glycohemoglobin. Motor nerve conduction studies were conducted on peroneal, ulnar, tibial, and median. Muscle biopsies taken from five patients and stained with hematoxylin and eosin.	1. Perioral fasciculation with bulbar paresis, elevated creatine kinase levels, hand tremor, hyporeflexia, and limb weakness with wasting were most consistent clinical findings. 2. Half of cases suffered from gynecomastia, sensory abnormalities, and family history of SBMA. 3. Weak correlation found between CAG repeat number and age of onset of paresis (*r* = −0.4). Age of onset of other symptoms and CAG repeat number were not significantly correlated. 4.CAG repeat length, 45–52.	Authors did not provide any strengths or weaknesses.	Authors recommended that future studies of a prospective nature should involve larger numbers of patients in order to better characterize natural progression of disease.
Atsuta et al., (2006), Natural history of spinal and bulbar muscular atrophy (SBMA): a study of 223 Japanese patients.	Investigate natural course of SBMA as assessed by 9 ADL milestones in 223 Japanese SBMA patients, and correlate this with age of onset of specific milestones during course of disease with the CAG-repeat number in AR gene.	1. Quantitative longitudinal retrospective. 2. Japanese males between 30 and 87 years. 3. 223 male patients. 4. Japan	Clinical course of SBMA reviewed in 223 of 303 patients. PCR amplification of CAG repeats was conducted. The size of the CAG repeat was analyzed on Fraglys software version 2.2, compared with co-electrophoresed PCR standards with known repeat sizes which were described previously by Doyu et al. (1992). Patients followed between 1 and 20 years. Initial onset and onset of nine activities of daily living (ADLs) (hand tremor, muscular weakness, requirement of a handrail, dysarthria, dysphagia, use of a cane, use of a wheelchair and development of pneumonia) were assessed. Age at death and cause of death also investigated. Modified Rankin scale used to assess clinical disability and serum levels of creatine kinase, AST, ALT, total cholesterol and HbA1c were compared to controls. Pearson's correlation coefficient was used where *p* < 0.05 was statistically significant.	1. Ages at onset of each ADL milestone strongly correlated with number of CAG repeats in AR gene. Inverse correlation between onset of muscle weakness and CAG repeat number (R = -0.483, *p* < 0.001). 2. CAG-repeat number did not correlate with time intervals between each ADL milestone. 3. Levels of serum testosterone were maintained at relatively high levels even at advanced ages. 4. CAG repeat range of 40–57, with mean of 46.6 ± 3.5.	Authors mentioned large sample size and employment of marked and apparent ADL milestones that patients recognized easily was a strength. Weaknesses included its retrospective structure, unsuccessful and its prospective assessment of individual patients. The decline curve was not successively and prospectively assessed in individual patients.	Authors recommended long-standing prospective studies to assess disease progression more accurately.
Ni et al., (2015), Genotype-phenotype correlation in Chinese patients with spinal and bulbar muscular atrophy	Elucidate genotype-phenotype correlation among Chinese SBMA patients.	1. Quantitative observational. 2. Males from Han Chinese population. 3. 155 SBMA males. 4. China	PCR amplification of CAG repeats. Prior to medication intervention serum creatine kinase (CK) was measured in 86 patients, and AST and lactic dehydrogenase (LDH) also measured in 32 and 44 patients respectively. Blood total cholesterol (TC), low-density lipoprotein (LDL) and triglyceride (TG) measured in 29 patients. 86 patients underwent EMG and NCS, Pearson's correlation coefficients used to assess relationship between CAG repeat number and other factors. Analysis of covariance (ANCOVA) and Bonferroni correction applied to neurophysiological values.	1. Inverse correlation between number of CAG repeats and age of onset of muscle weakness R^2 = 0.34, *p* < 0.0001. 2. Average CAG repeat length was 48.6 ± 3.5. 3. Inverse correlation between CK level and disease duration, and age at examination (*p* = 0.019 and *p* = 0.004, respectively). In addition, all nerve conduction findings, except the amplitudes of median nerve compound motor action potential, were positively correlated with CAG repeat number. 4. Average number of CAG repeats was 48.6 ± 3.5 (42–61), with average age of onset of muscle weakness 44.2 ± 10.2 (24–71) years.	Authors did not provide any strengths or weaknesses.	Authors recommended conducting a larger prospective study to better characterize t genotype-phenotype correlation among SBMA patients.
Song et al.*,* (2015), Clinical characteristics and genotype-phenotype correlation of Korean patients with spinal and bulbar muscular atrophy	Genotype-phenotype comparison of clinical and genetic data from Korean SBMA and control patients from different ethnicities.	1. Quantitative observational. 2. Korean male patients aged 20–71. 3. 40 male patients with SBMA. 4. Korea	Assessment of nine activities of daily living, including age of onset of symptoms and rate of disease progression. Number of CAG repeats verified using PCR. Genotype-phenotype correlations between CAG repeat number and clinical measurements made and analyzed using Spearman's Rank correlation tests with calculations performed on SPSS where *p* < 0.5 was statistically significant.	1. Significant inverse correlation between number of CAG repeats and age of onset of symptoms, including muscle weakness (*r* = −0.407, *p* = 0.009). 2. Median ages of onset and age at diagnosis were 44.5 and 52.5 years, respectively, and median number of CAG repeats was 44 (39.0–55.0). 3. Median interval between median rate of disease progression and onset and diagnosis were 0.23 score/year and 5.0 years respectively.	One weakness was reliance of some data upon patients' subjective memory. To their knowledge this was largest cohort of SBMA patients studied in Korea and is most likely to represent Korean patients with SBMA.	Large-scale cohort studies with various ethnicities needed.

## Appendix A Appendix

### A.1 Pubmed (MEDLINE) search terms

(“Bulbo-Spinal Atrophy, X-Linked” [Mesh] OR “spinal bulbar muscular atrophy”[tw] OR “kennedy's disease”[tw] OR “bulbospinal muscular atrophy”[tw]) AND ((“Muscle Weakness/diagnosis”[Mesh] OR “Muscle Weakness/etiology”[Mesh] OR “Muscle Weakness/genetics”[Mesh] OR “Muscle Weakness/pathology”[Mesh] OR “Muscle Weakness/physiology”[Mesh] OR “Muscle Weakness/physiopathology”[Mesh] OR “Muscle Weakness/prevention and control”[Mesh]) OR “muscle weakness”[tw] OR weak*[tw] OR paresis[tw] OR symptom*[tw]) AND (“age of onset”[tw] OR onset[tw] OR “age at onset”[tw]) AND (“Trinucleotide Repeat Expansion”[Mesh] OR “CAG repeat”[tw]) NOT (Hunting*[tw] OR “Huntington's disease”[tw] OR “Fragile X"[tw] OR “myotonic dystrophy”[tw] OR “amyotrophic lateral sclerosis”[tw] OR “ALS”[tw] OR “spinocerebellar ataxia”[tw] OR “oculopharyngeal muscular dystrophy”[tw]) .

### A.2 Web of science search terms

(“Bulbo-Spinal Atrophy, X-Linked” OR “spinal bulbar muscular atrophy” OR “kennedy's disease” OR “bulbospinal muscular atrophy”) AND (“Muscle weakness” OR weak* OR paresis OR symptom*) AND (“age of onset” OR onset) AND (“Trinucleotide Repeat Expansion” OR “CAG repeat”)

### A.3 SCOPUS Search terms

(ALL ((“Bulbo-Spinal Atrophy, X-Linked” OR “spinal bulbar muscular atrophy” OR “kennedy's disease” OR “bulbospinal muscular atrophy”) AND (“Muscle weakness” OR weak* OR paresis OR symptom*) AND (“age of onset” OR onset) AND (“Trinucleotide Repeat Expansion” OR “CAG repeat”)))

### A.4 Cambridge University Press search terms:

“SBMA”.

### A.5 Annals of Neurology - Wiley Online Library search terms

“Manabu Doyu” AND “spinal bulbar muscular atrophy”.
